# A rare case of incidental finding of GIST during sleeve gastrectomy: Case report

**DOI:** 10.1016/j.ijscr.2019.10.040

**Published:** 2019-11-01

**Authors:** Kusay Ayoub, Aghyad Kudra Danial, Ahmad Sankari Tarabishi, Baraa Shebli, Mohammed Yasser Halwani, Nihad Mahli

**Affiliations:** a Department of Surgery, Aleppo University Hospital, University of Aleppo, Syria; b University of Aleppo, Faculty of Medicine, Syria

**Keywords:** Bariatric surgery

## Abstract

**Introduction:**

Gastrointestinal stromal tumors (GISTs) is the term used to describe rare stromal neoplasms that are located in the gastrointestinal tract, it most commonly arises in the stomach.

GIST is usually asymptomatic and discovered incidentally by Computed Tomography (CT) or Endoscopy.

**Presentation of case:**

We report a case of incidental finding of GIST during Sleeve gastrectomy in a 56 year-old female that presented with a complaint of sever obesity and articular pain in lower limbs.

The tumor was not compromising the performance of a save vertical gastric resection, so the resection was done and a mass specimen was sent for pathological examination that confirmed Gist with a low grade of malignancy.

**Discussion and conclusion:**

As a conclusion, we recommend keeping in mind incidental finding of GIST during abdominal surgeries and especially Sleeve gastrectomy, in order to detect this tumor as earlier as possible and have a good prognosis with a low chance for recurrence.

## Highlights

• Gastrointestinal stromal tumors (GISTs) is the term used to describe rare stromal neoplasms that are located in the gastrointestinal tract.
• GIST could be found incidental during abdominal surgeries and especially Sleeve gastrectomy.
• GISTs are usually asymptomatic and discovered incidentally by Computed Tomography (CT) or Endoscopy.
• Surgical excision is performed for the majority of patients.

## 1 Introduction

Gastrointestinal stromal tumors (GISTs) is the term used to describe rare stromal neoplasms that are located in the gastrointestinal tract. Nearly two thirds occur in the stomach, 30% in the small intestine, and 10% elsewhere [1]. Recent studies revealed that the cellular origin of GISTs is the interstitial cell of Cajal, which is an intestinal pacemaker responsible for peristaltic contractions [2].

By immunohistochemical test, most GISTs stain positive for c-kit (CD117) and CD34, and malignant GISTs show an increased cell division rate [1].

The incidence of GIST is approximately 15 cases per million population a year, which in the United States equals 5000 cases per year [3]. In most cases GIST is asymptomatic and usually discovered incidentally by Computed Tomography (CT) or Endoscopy [4]. The treatment of choice for GIST is surgery for small, local, and non-metastatic tumors and surgery with combination of Imatinib for recurrent or metastatic GIST [5].

Imaging studies for GIST include CT scan for patients with suspected abdominal mass, magnetic resonance imaging MRI and positron emission tomography PET [5].

In this case, we present an incidentally discovered GIST during sleeve gastrectomy in a 56 year-old female.

This work has been reported in line with the SCARE criteria [6].

## 2 Case presentation

A 56 year-old female presented to department of Bariatric surgery complaining of sever obesity and articular pain in lower limbs, her weight was 110 kg, Height 167 cm and Body Mass Index (BMI) was 39.44 kg/m^2^. As a history she has Diabetes Mellitus (DM) that complicated prior with a left hemi foot amputation.

As she is a diabetic patient, she was on Insulin after failure of oral hypoglycemic medications.

The patient had previously conservative treatment for obesity with Orlistat, which had no effective impact on her weight.

Her TSH and Serum Cortisol values were normal.

Therefore, the patient was advised to operate surgery because of her high BMI and failure of obesity conservative treatment.

Through surgery preparation, her lab tests were normal, upper gastrointestinal endoscopy UGI and abdominal ultrasound both had revealed no abnormal findings.

**Fig. 1:**
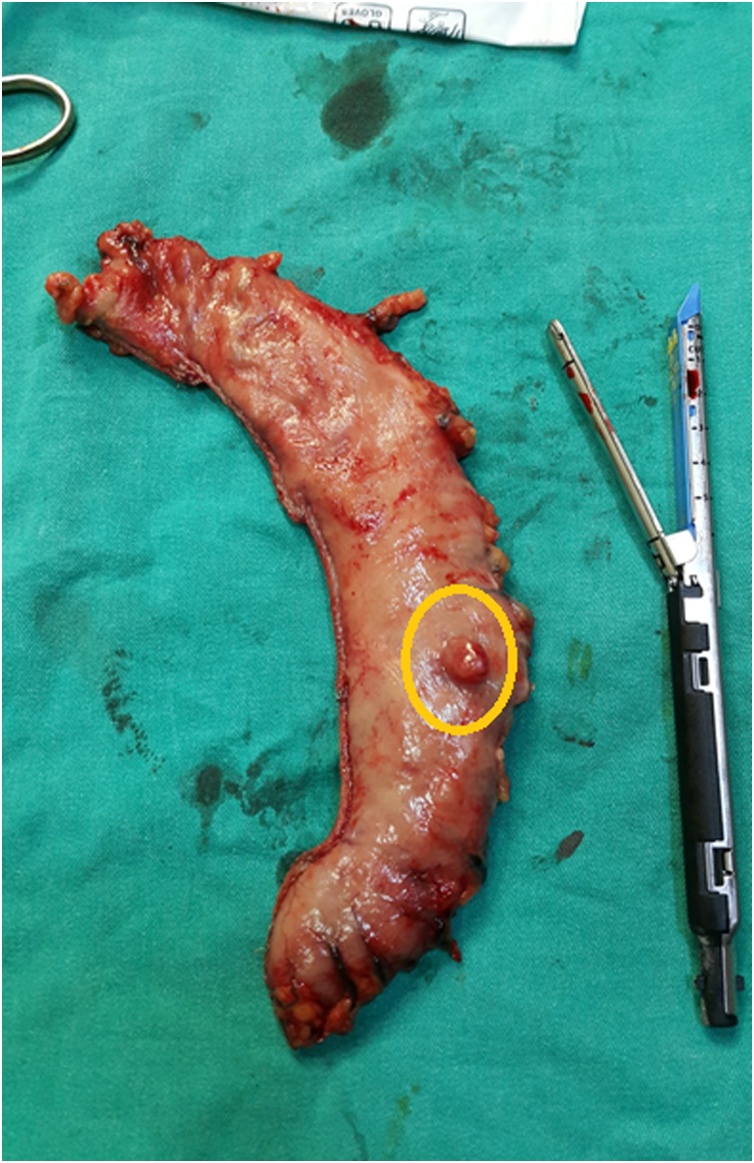
An incidental small rounded mass less than 2 cm discovered at the anterior aspect of the stomach.

We operated the patient by abdominal laparoscopy with sleeve gastrectomy, at the beginning of procedure, we discovered an incidental rounded, less than 2 cm small mass on the anterior aspect of the stomach (Fig. 1), it was not compromising the performance of a save vertical gastric resection, so the resection was done and a mass specimen was sent for pathological examination.

**Fig. 2:**
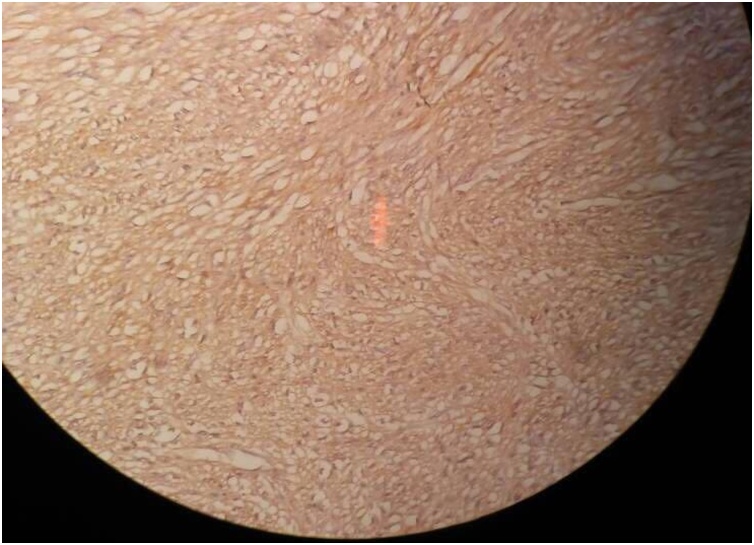
1.3 mm in diameter mass, comprised of spindle cells with minimal nuclear pleomorphism and a few mitotic activity (mitotic index 1–2 × 10 high power fields).

Pathology report revealed 1.3 mm in diameter mass, comprised of spindle cells with minimal nuclear pleomorphism and a few mitotic activity (mitotic index 1–2 × 10 high power fields) (Fig. 2) and suggested a gastrointestinal stromal tumor (GIST) with a low grade of malignancy.

The tumor invaded the gastric wall, penetrated the submucousal layer and reached the mucous layer without infiltrating it.

Through immunologic examination, all tumor cells reacted positively to CD117 with *Dako EnVision* system.

After surgery, the patient is in a good condition, she lost 25 kg through 3 months, her fasting blood sugar is around normal (100–130 mg/dl), and the tumor required no additional treatment.

## 3 Discussion

GIST are rare tumors of the gastrointestinal tract, accounting for less than 1% of all gastrointestinal tract tumors, it most commonly arises in the stomach [7,8].

GIST is usually asymptomatic and discovered incidentally by Computed Tomography (CT) or Endoscopy [4].

The most common symptom at presentation of GIST patients is bleeding [9].

Recently, with the development of laparoscopy especially in the field of Bariatric surgery, incidental findings of GISTs that have not presented yet occurred. Sanchez et al. reported the incidence of incidental finding of a pathology during laparoscopic Bariatric surgery of 2% [10].

Many studies reported the incidental finding of GIST in obese patients undergoing laparoscopic sleeve gastrectomy.

Beltran et al. reported the first case of an incidental GIST finding during laparoscopic sleeve gastrectomy in 2009 [11].

Crouthamel et al. reported the incidental finding of 12 GISTs in 1415 patients undergoing sleeve gastrectomy procedures with an incidence of 0.8% [11].

Chiappetta et al. reported the incidental finding of 8 GISTs in 2603 patients undergoing Bariatric surgery with an incidence of 0.31% [12].

Patients with incidental finding of GIST in the two latest studies were disease-free upon follow-up due to the early detection of these tumors. The numbers in both studies indicate that GIST is an uncommon incidental finding in Bariatric surgery, including sleeve gastrectomy, but it is an important finding due to the early detection of these tumors which can alter the prognosis.

Chaippetta et al. reported the use of upper endoscopy for evaluation before surgery, but none of the GISTs were diagnosed by upper endoscopy because the tumor was located at the serousal surface of the stomach [12].

Our patient did not have any symptoms of GIST rather she presented with the complaint of morbid obesity. She had diabetes mellitus which is reported to increase the incidence and recurrence of GIST [13]. This early detection of the tumor may have improved the prognosis significantly, especially as she had diabetes mellitus.

Since the treatment of choice for GIST is R0 resection (margin-negative) [12] and the chance for recurrence is 0% in low-grade GIST [14,15], it is very important to detect these tumors as early as possible and one should always keeping them in mind in abdominal surgeries.

## Funding

We have no funding sources.

## Ethical approval

No ethical approval is needed for this kind if studies in our institution.

## Consent

Written consent was obtained from the patient.

## Author contribution

AKD and NM performed the surgery to the patient and collected the data. AST, BS and MYH drafted the first article. AKD, AST, BS and MYH did the final revision.

## Registration of research studies

NA.

## Guarantor

Dr, Kusay Ayoub.

## Provenance and peer review

Not commissioned, externally peer-reviewed.

## Declaration of Competing Interest

We have no conflict of interest.
